# Supplementary material for: Aedes aegypti Argonaute 2 controls arbovirus infection and host mortality
Source: Nat Commun. 2023 Sep 18;14:5773. doi: 10.1038/s41467-023-41370-y (PMC10507101; doi:10.1038/s41467-023-41370-y)

Supplementary Information

Aedes aegypti Argonaute 2 controls arbovirus infection and host mortality

Shengzhang Dong & George Dimopoulos

W. Harry Feinstone Department of Molecular Microbiology and Immunology, Bloomberg School of Public Health, Johns Hopkins University, 615 N. Wolfe Street, Baltimore, MD 21205-2179, USA.

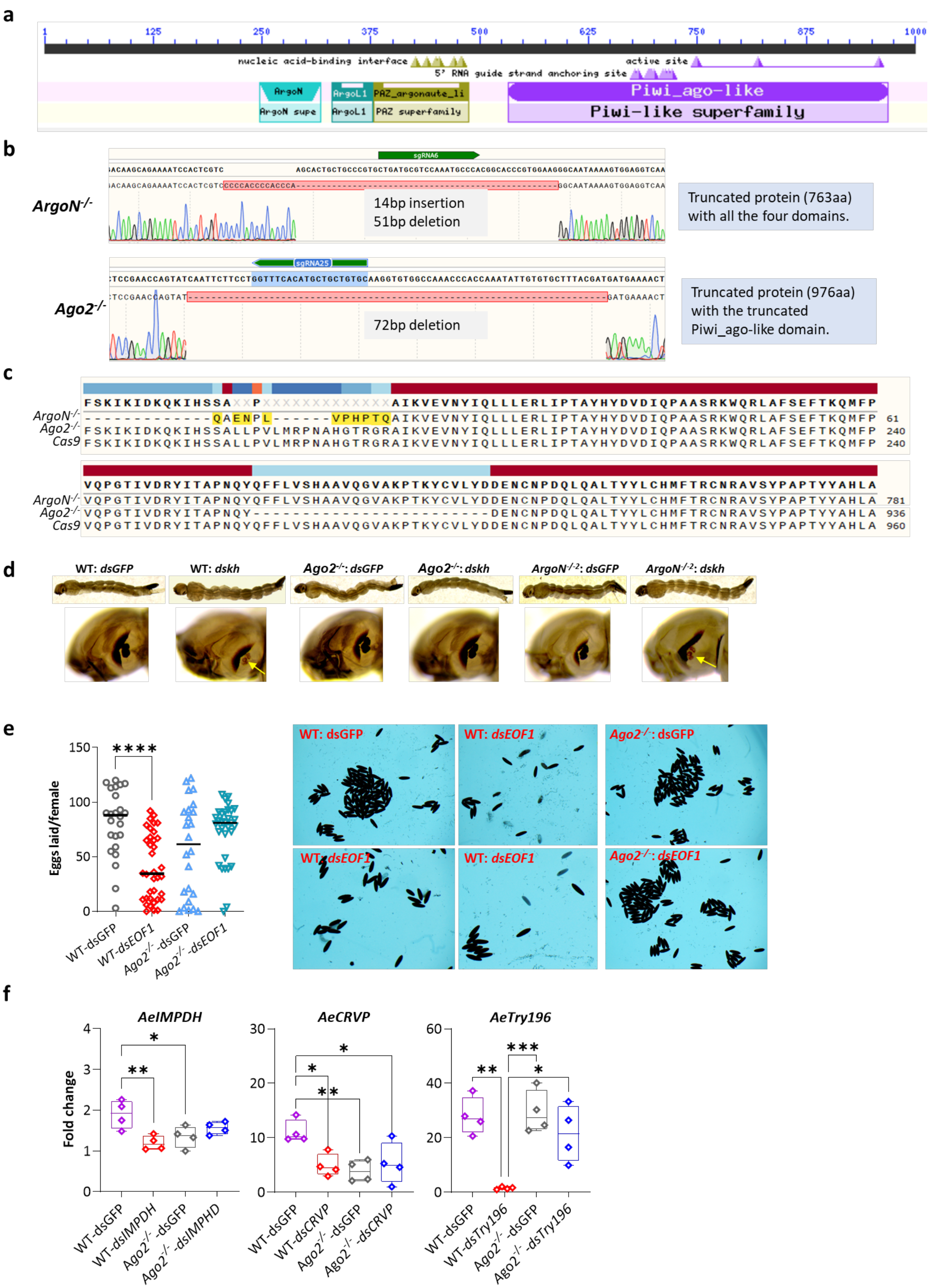

a
nucleic acid-binding interface
5' RNA guide strand anchoring site
active site
ArgoN
ArgoN supe
ArgoL1
PAZ_argonaute_li
PAZ superfamily
Piwi_ago-like
Piwi-like superfamily
b
sgRNA6
ArgoN-/-
14bp insertion
51bp deletion
Truncated protein (763aa) with all the four domains.
sgRNA25
Ago2-/-
72bp deletion
Truncated protein (976aa) with the truncated Piwi_ago-like domain.
c
ArgoN-/-
Ago2-/-
Cas9
d
WT: dsGFP
WT: dskh
Ago2-/-: dsGFP
Ago2-/-: dskh
ArgoN-/-2: dsGFP
ArgoN-/-2: dskh
e
Eggs laid/female
****
WT-dsGFP
WT-dsEOF1
Ago2-/- -dsGFP
Ago2-/- -dsEOF1
WT: dsGFP
WT: dsEOF1
Ago2-/-: dsGFP
WT: dsEOF1
WT: dsEOF1
Ago2-/-: dsEOF1
f
AeIMPDH
AeCRVP
AeTry196
Fold change
WT-dsGFP
WT-dsIMPDH
Ago2-/- -dsGFP
Ago2-/- -dsIMPHD
WT-dsGFP
WT-dsCRVP
Ago2-/- -dsGFP
Ago2-/- -dsCRVP
WT-dsGFP
WT-dsTry196
Ago2-/- -dsGFP
Ago2-/- -dsTry196

Supplementary Fig.1 Characterization of the *Ago2* knockout mutant lines. a, functional domains of Ago2 predicted using the conserved domain search from the NCBI. **b,** sequencing trace data showing indels, and differences in the DNA and protein sequences of *Ago2* DNA in *Ago2* knockout mutant lines. **c**, amino acid sequence mutations in the *Ago2* knockout mutant lines. **d**, phenotypes of 3rd instar larvae hatched from *kh* dsRNA-injected eggs of WT, *Ago2*$^{-/-}$ and *AgoN*$^{-/-}$ mutants. Larvae hatched from GFP dsRNA-injected eggs were used as a control. Yellow arrows indicate defects in eye pigment. **e**, eggs laid from *EOF1* dsRNA-injected females of WT and *Ago2*$^{-/-}$ mutants. *P*-values were determined by using an unpaired two-sided *t*-test between dsGFP- and *EOF1* dsRNA-injected mosquitoes. n=2 biological replicates. **f**, silencing efficiency of *AeIMPDH*, *AeCRVP* and *AeTry196* dsRNA injected WT and *Ago2*$^{-/-}$ mutants at 3 days post dsRNA injection. n=4 biological replicates. Statistical significance was determined by a one-way ANOVA. *P<0.05, **P<0.01, ***, P< 0.001, ****P<0.0001. Source data are provided as a Source Data file.

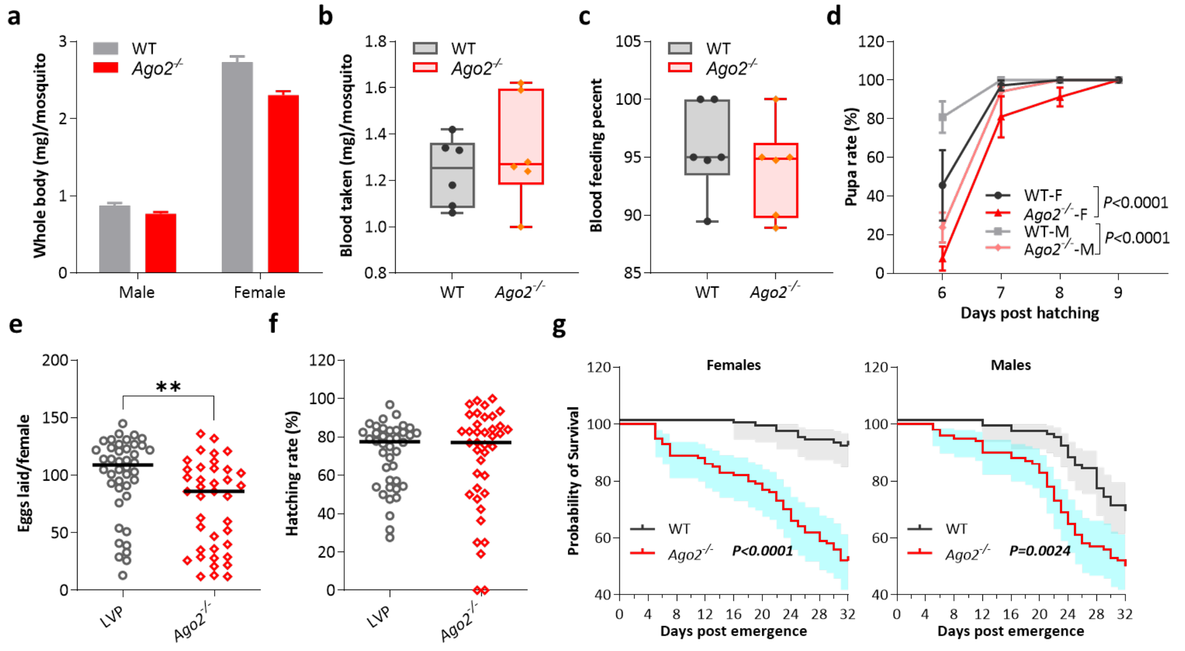

Supplementary Fig.2 Fitness test of WT and *Ago2*$^{-/-}$ mutants. a, the whole-body weight of one-day-old females and males measured by an analytical balance. n=6 biological replicates. **b**, the amount of blood taken by one-week-old individual female. The amount of blood take by each female was calculated by subtracting the body weight of pre-bloodfed individuals. n=6 biological replicates. **c**, blood-feeding propensity of females. n=6 biological replicates. **d**, pupation rate at different days post hatching. n=6 biological replicates. **e**, number of eggs laid by individual female. **f**, hatching rate of eggs. n=48 biological samples. Statistical significance between WT and *Ago2*$^{-/-}$ mutants was determined by using an unpaired two-sided *t*-test (**a-c**), two-way ANOVA with multiple comparisons (**d**), and an unpaired two-sided Mann-Whitney test (**e**, **f**). **$P<0.01$, ****$P<0.0001$. **g**, longevity of males and females maintained on a 10% sterile sucrose. Data are represented as percents ± SE (n=5 biological replicates). *P*-values were determined by using the logrank (Mantel-Cox) test. Source data are provided as a Source Data file.

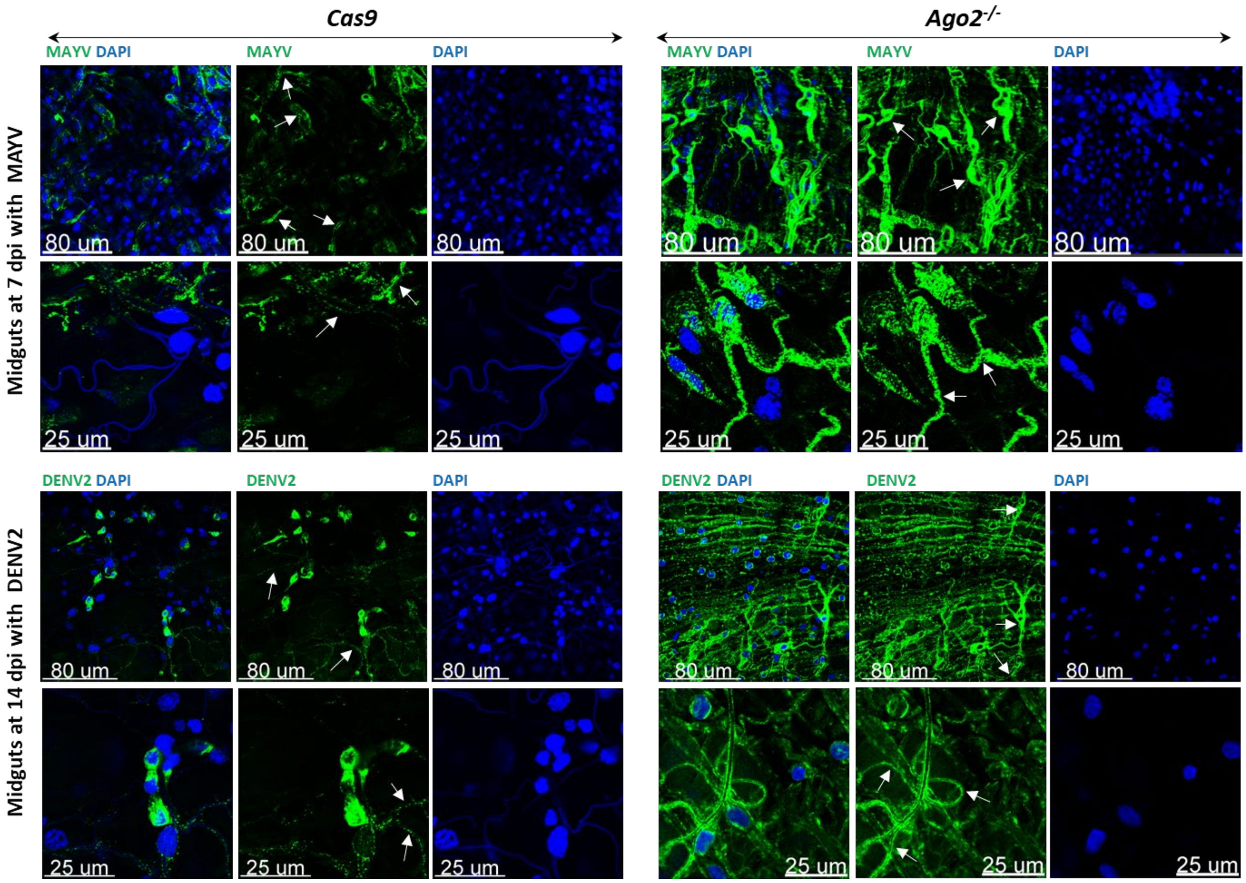

Supplementary Fig.3 IFA detection of MAYV and DENV2 antigen in midguts and midgut-associated tracheas of *Ago2*$^{-/-}$ and *Cas9* females at 7 days post infection (dpi) and 14 dpi, respectively. MAYV and DENV2 were detected with their corresponding monoclonal antibody (green). Nuclei were stained with DAPI (blue). White arrows indicate tracheas. The IFA images are representative of three biologically independent samples.

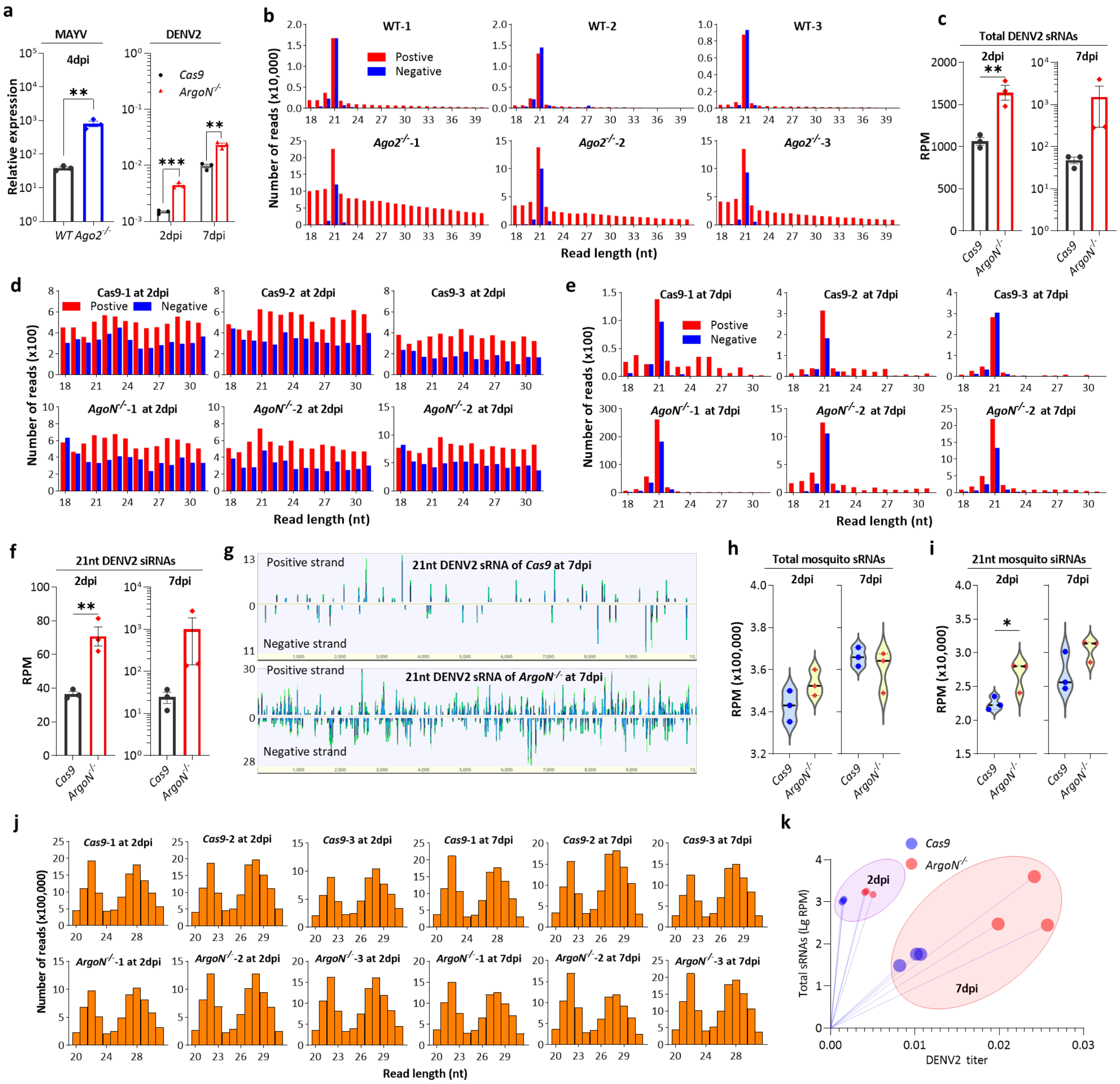

Supplementary Fig.4 Impact of *Ago2* disruption on production of MAYV-, DENV2- and mosquito-derived small RNAs . a, relative expression ($2^{-\Delta CT}$) of DENV2 or MAYV to ribosome *S7* in *Ago2* knockout mutants and the control mosquitoes at different days post DENV2 or MAYV infection. **b,** length distribution of sRNAs mapping to the positive (red bars) and negative (blue bars) strand of the MAYV genome in WT and *Ago2*$^{-/-}$ mutants at 4 dpi with MAYV. **c,** total number (reads per million mapped reads, RPM) of DENV2 sRNAs in *ArgoN*$^{-/-}$ mutants and the parental line (*Cas9*) at 2- and 7-days post-infection (dpi) as determined by sRNA sequencing. **d-e**, length distribution of sRNAs mapping to the positive (red bars) and negative (blue bars) strand of the DENV2 genome in *ArgoN*$^{-/-}$ mutants and *Cas9* mosquitoes at 2 dpi (**d**) and 7 dpi (**e**). **f**, number of 21-nt siRNAs mapped to the DENV2 genome. **g**, distribution of 21-nt DENV2 siRNA reads in positive and negative strand of the DENV2 genome and the relative abundances of siRNAs. Number of total sRNA (**h**) and 21-nt siRNAs (**i**) mapped to the *Ae. aegypti* genome in *ArgoN*$^{-/-}$ mutants and *Cas9* at 2 and 7 dpi with DENV2. **j**, length distribution of the mosquito

sRNAs. **k**, correlation between the abundance of total DENV2 sRNAs and titer of DENV2 in *ArgoN*$^{-/-}$ mutants and *Cas9* mosquitoes as determined by principal component analysis (PCA). *P*-values were determined by using an unpaired two-sided *t*-test (n=3). **P*<0.05, ***P*<0.01. Source data are provided as a Source Data file.

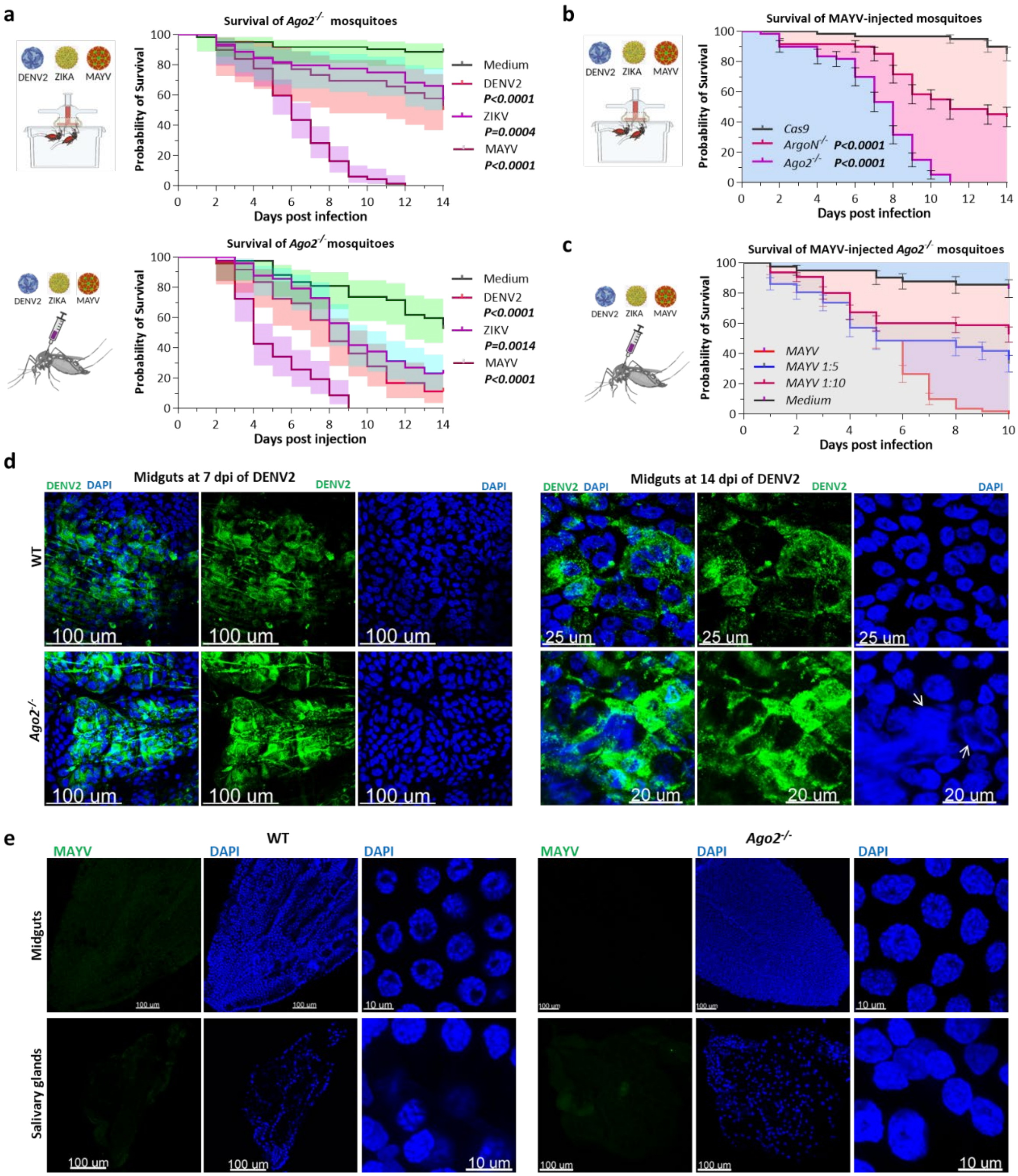

Supplementary Fig.5 Effect of *Ago2* disruption on mosquito death upon arbovirus infection. a, survival curves of *Ago2*$^{-/-}$ females that ingested a blood meal containing DENV2, ZIKV, or MAYV, or C6/36 cell culture medium as a control (Mock) or were injected with DENV2, ZIKV, MAYV, or control medium. The error bands were indicated by the shaded region. **b**, survival curves for *Ago2*$^{-/-}$, *ArgoN*$^{-/-}$, and *Cas9* (the parental line) females that ingested a blood meal containing MAYV. **c**, survival curves of *Ago2*$^{-/-}$ mutants that were injected with different titers of MAYV or control medium. Data are represented as percents ± SE (n=3 biological replicates). *P* values between mutants and *Cas9* mosquitoes were determined using the logrank (Mantel-Cox) test. **d**, IFA detection with the anti-DENV2 monoclonal

antibody (green) of DENV2 in midguts of *Ago2*$^{-/-}$ and WT females at 7 days post-infection (dpi) and 14 dpi. Nuclei were stained with DAPI (blue). White arrows indicate abnormal nuclei. **e**, IFA detection of midguts and slavery glands in uninfected WT and *Ago2*$^{-/-}$ mutants. Midguts and salivary glands were dissected from 7-days and 14 days post blood feeding, respectively. Green channel indicates anti-MAYV monoclonal antibody staining, and nuclei were stained with DAPI (blue). The IFA images are representative of three biologically independent samples. Source data are provided as a Source Data file.

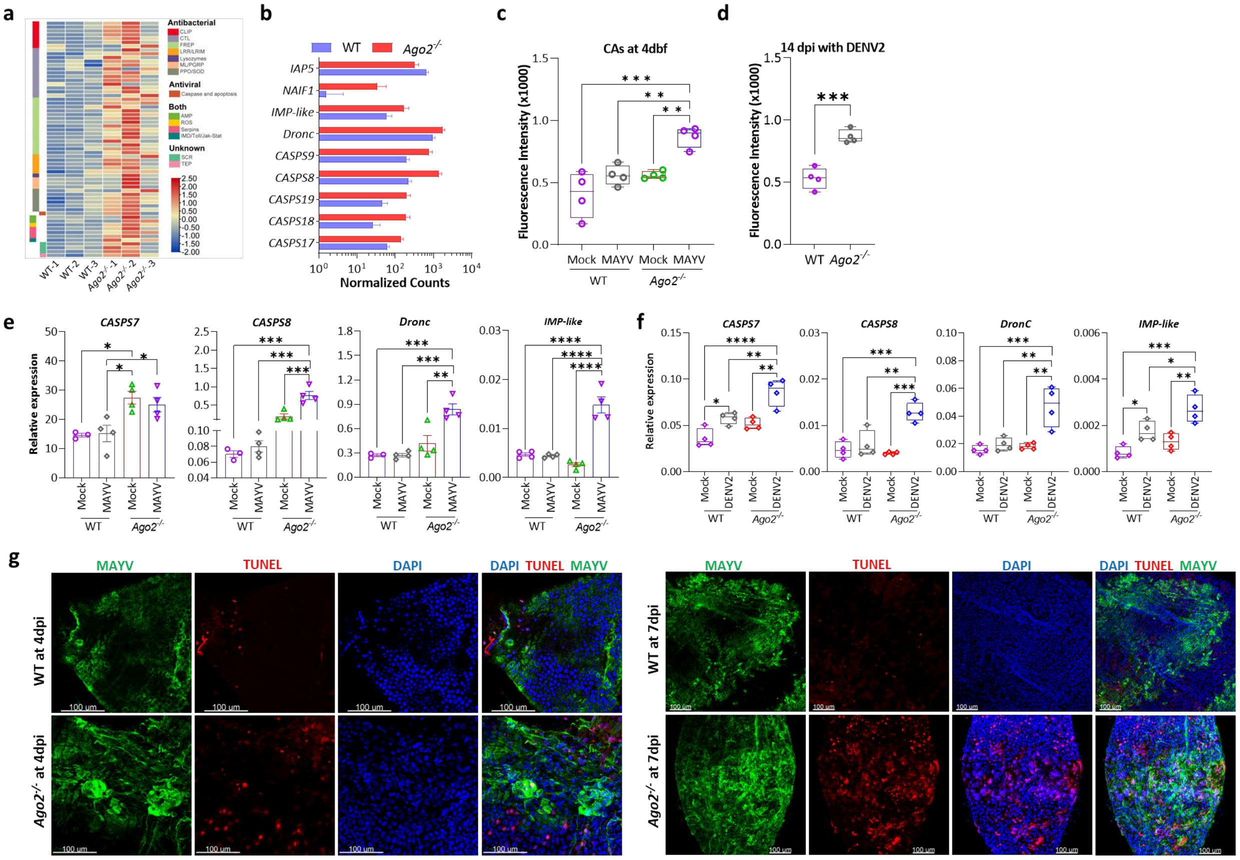

Supplementary Fig.6 Analysis of differentially expressed immune genes between uninfected *Ago2*$^{-/-}$ mutants and WT and arbovirus infection induced apoptosis in *Ago2*$^{-/-}$ mutants. a, heatmap showing the upregulated immunity genes in uninfected *Ago2*$^{-/-}$ mutants as compared to the WT. **b**, significantly regulated caspase and apoptosis genes in the transcriptome of *Ago2*$^{-/-}$ mutants. **c**, caspase activity in the midguts of *Ago2*$^{-/-}$ and WT females at 4 dpi with MAYV as determined by the EnzChek caspase-3 assay. **d**, caspase activity in *Ago2*$^{-/-}$ mutants and WT mosquitoes at 14 dpi with DENV2. **e**, expression of caspase and apoptosis genes in the carcasses of *Ago2*$^{-/-}$ and WT females at 4 dpi with MAYV as determined by qPCR. **f**, confirmation by qPCR of the upregulated expression of caspase and apoptosis genes in midguts of *Ago2*$^{-/-}$ mutants at 14 dpi with DENV2. **g**, apoptotic cells (red) in the midguts of *Ago2*$^{-/-}$ and WT females as determined by TUNEL assay. MAYV infection is indicated in green and nuclei in blue. n=4 biological replicates. n=4 biological replicates (**c**-**f**). Statistical significance between WT and *Ago2*$^{-/-}$ mutants was determined by one-way ANOVA (**c**, **e** and **f**) and an unpaired two-sided *t*-test (**d**). **P*<0.05, ***P*<0.01, ***, *P*< 0.001, *****P*<0.0001. The IFA images are representative of three biologically independent samples. Source data are provided as a Source Data file.

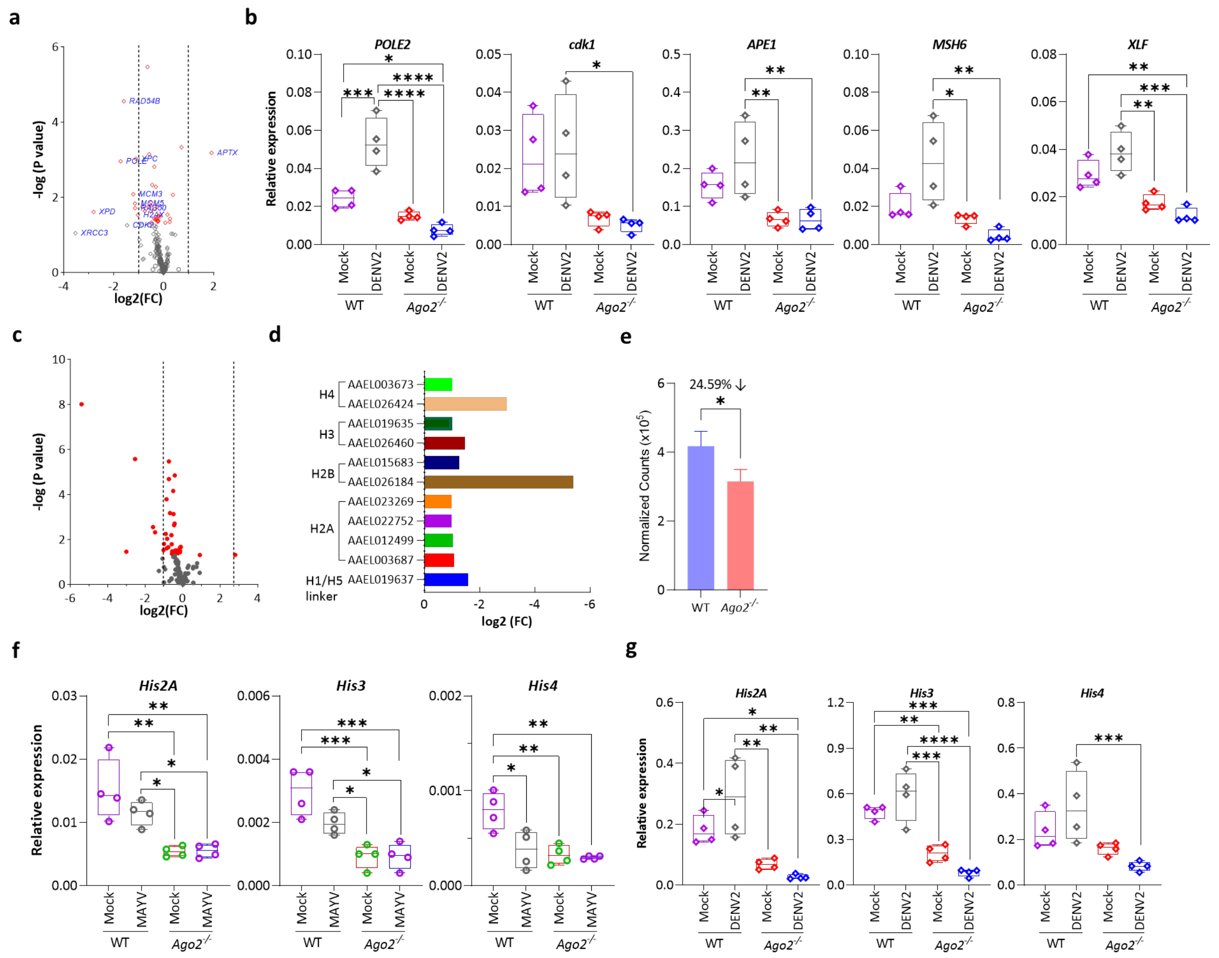

Supplementary Fig.7 Analysis of histone gene and DNA repair gene (DRG) expression in *Ago2*$^{-/-}$ mutants. a, volcano plot illustrating fold changes of mRNAs corresponding to genes with predicated function in DNA damage response in uninfected *Ago2*$^{-/-}$ mutants as compared to WT at 4 days post blood feeding. **b**, confirmation by qPCR of the downregulated DRGs in *Ago2*$^{-/-}$ mutants at 14 dpi with and without DENV2 infection. **c**, volcano plot illustrating fold changes in the mRNA abundance of genes related to histones in the transcriptome of uninfected *Ago2*$^{-/-}$ mutants as compared to the WT. **d,** significantly downregulated (fold change≥2) histone genes in the transcriptome of uninfected *Ago2*$^{-/-}$ mutants. **e**, overall level of histone mRNA abundance in the transcriptome of uninfected *Ago2*$^{-/-}$ mutants and WT mosquitoes. qPCR detecting the expression of histone genes in the midguts of *Ago2*$^{-/-}$ mutants and WT mosquitoes at 4 dpi with or without MAYV (**f**) and 14 dpi with or without DENV2 (**g**). Statistical significance WT and *Ago2*$^{-/-}$ mutants was determined by a one-way ANOVA for **b**, **f** and **g** (n=4 biological replicates), an unpaired two-sided *t*-test for **e** (n=3 biological replicates). *P<0.05, **P<0.01, ***, P< 0.001, ****, P< 0.0001. Source data are provided as a Source Data file.

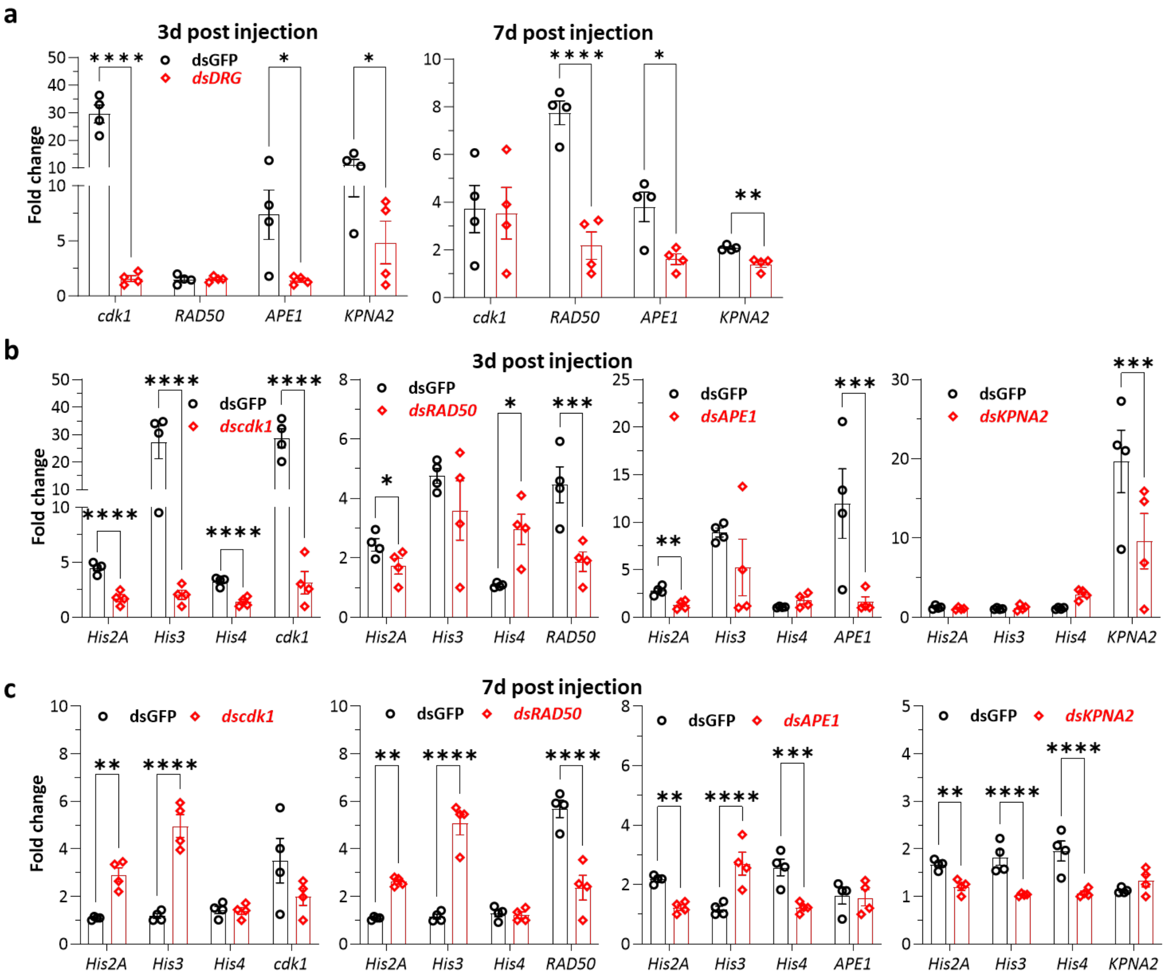

Supplementary Fig.8 Effect of DNA repair gene (DRG) silencing on histone expression. a, silencing efficiency of *DRG* in WT mosquitoes that were injected with *DRG* dsRNA (a mix of dsRNAs of *cdk1*, *RAD50, APE1* and *KPNA2*, each 3µg/µl) at 3- and 7- days post injection. **b** and **c**, WT females were injected with 3µg/µl of ds*cdk1*, ds*RAD50,* ds*APE1* or ds*KPNA2*, and the expression of histone genes and silencing efficiency was monitored at 3 days (**b**) and 7 days (**c**) post dsRNA injection, respectively. GFP dsRNA was injected as a control. n=4 biological replicates. Statistical significance was determined by a two-way ANOVA with multiple comparison test (Fisher's LSD test) from the GraphPad. *P<0.05, **P<0.01, ***, P< 0.001, ****, P< 0.0001. Source data are provided as a Source Data file.

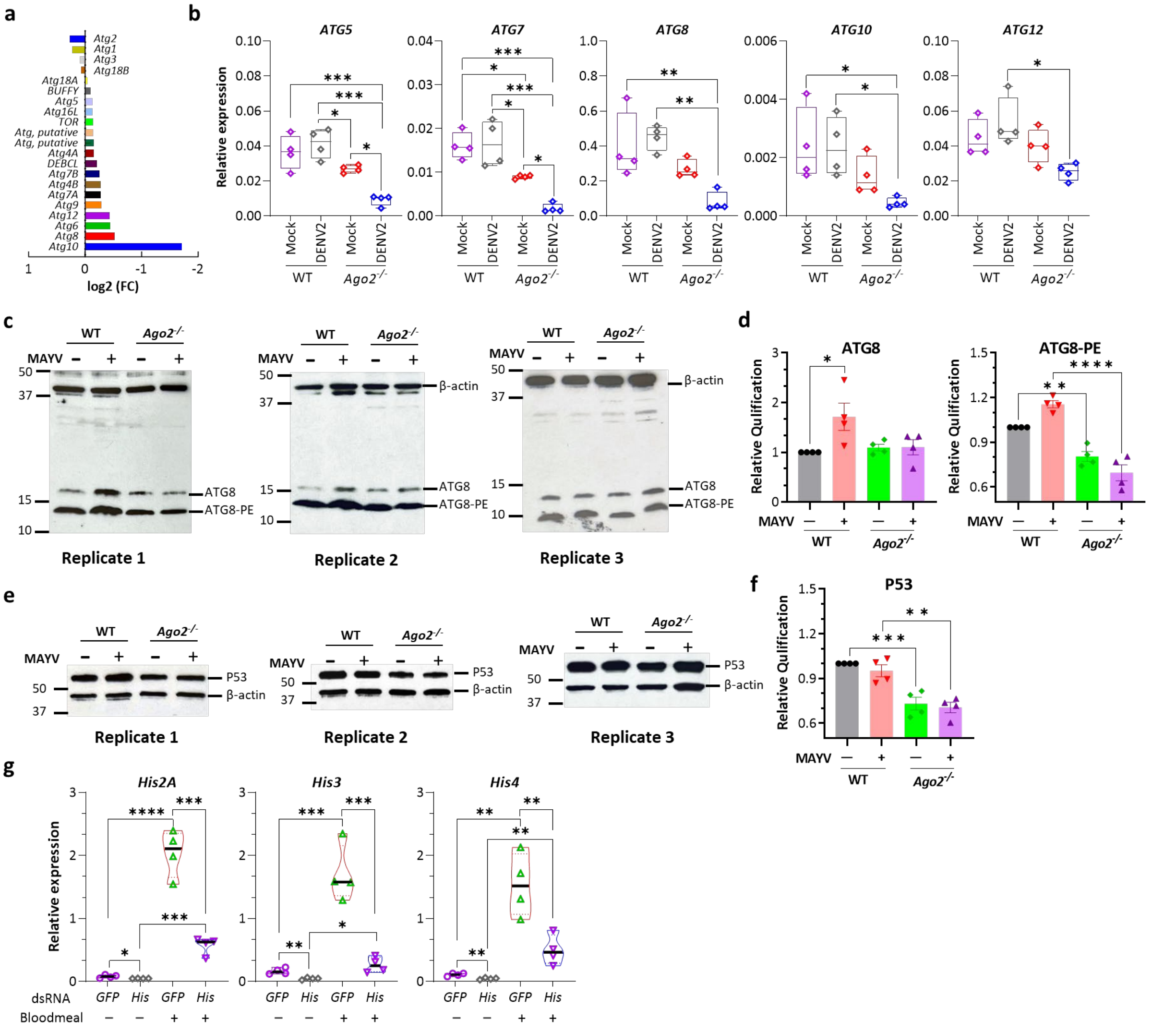

Supplementary Fig.9 Effect of *Ago2* disruption on expression of autophagy-related genes (*Atg*). a, fold change of autophagy-related gene mRNAs in the transcriptome of uninfected *Ago2*$^{-/-}$ mutants as compared to that of the WT mosquitoes at 4 days post blood feeding. **b**, expression of *Atg* genes in *Ago2*$^{-/-}$ mutants and WT mosquitoes at 14 days post-infection (dpi) with DENV2, as detected by qPCR. Western blots showing the protein levels of ATG8, ATG8-phosphatidylethanolamine (ATG8-PE) (**c**) and P53 (**e**), and relative protein abundance of ATG8, ATG8-PE (**d**) and P53 (**f**) normalized to the β-actin in *Ago2*$^{-/-}$ and WT females at 4 dpi with MAYV; β-actin was used as a loading control. **g**, silencing efficiency of histone genes in WT mosquitoes that were injected with a mixture of dsRNA from *H2A*, *H3,* and *H4*. dsRNA for *GFP* was injected as a control. The expression of *H2A*, *H3,* and *H4* at 3 days post-injection and 4 days post blood-feeding, as determined by qPCR. Statistical significance was determined by an unpaired two-sided *t*-test for **d and f** (n=3 biological replicates) and one-way ANOVA for **b** and **g** (n=4 biological replicates). *P<0.05, **P<0.01, ***, P< 0.001, ****, P< 0.0001. Source data are provided as a Source Data file.

Supplementary Table 1 Gene ontology (GO) of the upregulated genes in *Ago2*$^{-/-}$ mutants as compared to WT mosquitoes at 4 dpi with MAYV.

ID	Name	Bgd count	Result count	Pct of bgd	Fold enrichment	Odds ratio	P-value
GO:0006826	iron ion transport	7	4	57.1	8.5	18.59	0.000602
GO:0006879	cellular iron ion homeostasis	8	4	50	7.44	13.94	0.00114
GO:0007229	integrin-mediated signaling pathway	8	4	50	7.44	13.94	0.00114
GO:0055072	iron ion homeostasis	11	5	45.5	6.76	11.63	0.000445
GO:0046916	cellular transition metal ion homeostasis	9	4	44.4	6.61	11.15	0.001943
GO:0055076	transition metal ion homeostasis	13	5	38.5	5.72	8.72	0.001107
GO:0000041	transition metal ion transport	12	4	33.3	4.96	6.97	0.006486
GO:0009617	response to bacterium	15	5	33.3	4.96	6.98	0.002306
GO:0042742	defense response to bacterium	15	5	33.3	4.96	6.98	0.002306
GO:0045087	innate immune response	15	5	33.3	4.96	6.98	0.002306
GO:0010951	negative regulation of endopeptidase activity	22	7	31.8	4.73	6.53	0.000421
GO:0052548	regulation of endopeptidase activity	22	7	31.8	4.73	6.53	0.000421
GO:0006952	defense response	26	8	30.8	4.58	6.22	0.000211
GO:0006959	humoral immune response	10	3	30	4.46	5.97	0.02542
GO:0019730	antimicrobial humoral response	10	3	30	4.46	5.97	0.02542
GO:0019731	antibacterial humoral response	10	3	30	4.46	5.97	0.02542
GO:0006955	immune response	24	7	29.2	4.34	5.76	0.000759
GO:0009607	response to biotic stimulus	24	7	29.2	4.34	5.76	0.000759
GO:0043207	response to external biotic stimulus	24	7	29.2	4.34	5.76	0.000759
GO:0044419	biological process involved in interspecies interaction between organisms	24	7	29.2	4.34	5.76	0.000759
GO:0051707	response to other organism	24	7	29.2	4.34	5.76	0.000759
GO:0098542	defense response to other organism	24	7	29.2	4.34	5.76	0.000759
GO:0002376	immune system process	26	7	26.9	4	5.15	0.001283
GO:0010466	negative regulation of peptidase activity	26	7	26.9	4	5.15	0.001283
GO:0045861	negative regulation of proteolysis	26	7	26.9	4	5.15	0.001283
GO:0052547	regulation of peptidase activity	27	7	25.9	3.86	4.89	0.001633
GO:0006030	chitin metabolic process	104	26	25	3.72	4.76	3.17E-09
GO:0006873	cellular ion homeostasis	16	4	25	3.72	4.64	0.019239
GO:0006875	cellular metal ion homeostasis	16	4	25	3.72	4.64	0.019239
GO:0030003	cellular cation homeostasis	16	4	25	3.72	4.64	0.019239
GO:0055065	metal ion homeostasis	20	5	25	3.72	4.65	0.008997
GO:1901071	glucosamine-containing compound metabolic process	106	26	24.5	3.65	4.64	4.91E-09
GO:0006040	amino sugar metabolic process	108	26	24.1	3.58	4.52	7.50E-09
GO:0051346	negative regulation of hydrolase activity	29	7	24.1	3.59	4.45	0.002551
GO:0006022	aminoglycan metabolic process	116	27	23.3	3.46	4.33	8.47E-09
GO:0055082	cellular chemical homeostasis	18	4	22.2	3.31	3.98	0.029078
GO:0030162	regulation of proteolysis	32	7	21.9	3.25	3.91	0.004614
GO:0050801	ion homeostasis	23	5	21.7	3.23	3.87	0.016525
GO:0055080	cation homeostasis	23	5	21.7	3.23	3.87	0.016525
GO:0098771	inorganic ion homeostasis	23	5	21.7	3.23	3.87	0.016525

GO:0006836	neurotransmitter transport	24	5	20.8	3.1	3.67	0.019749
GO:0043086	negative regulation of catalytic activity	34	7	20.6	3.06	3.62	0.006562
GO:0016055	Wnt signaling pathway	21	4	19	2.83	3.28	0.048529
GO:0198738	cell-cell signaling by wnt	21	4	19	2.83	3.28	0.048529
GO:0044092	negative regulation of molecular function	37	7	18.9	2.81	3.26	0.010545
GO:0048878	chemical homeostasis	27	5	18.5	2.75	3.17	0.031791
GO:0032269	negative regulation of cellular protein metabolic process	49	9	18.4	2.73	3.15	0.004823
GO:0051248	negative regulation of protein metabolic process	49	9	18.4	2.73	3.15	0.004823
GO:0051172	negative regulation of nitrogen compound metabolic process	50	9	18	2.68	3.07	0.005539
GO:0009605	response to external stimulus	57	10	17.5	2.61	2.98	0.004283
GO:0006633	fatty acid biosynthetic process	35	6	17.1	2.55	2.89	0.027534
GO:0072330	monocarboxylic acid biosynthetic process	35	6	17.1	2.55	2.89	0.027534
GO:0007267	cell-cell signaling	43	7	16.3	2.42	2.71	0.023322
GO:0007155	cell adhesion	75	12	16	2.38	2.67	0.004044
GO:0022610	biological adhesion	75	12	16	2.38	2.67	0.004044
GO:0006631	fatty acid metabolic process	44	7	15.9	2.37	2.64	0.026182
GO:0006508	proteolysis	707	105	14.9	2.21	2.65	2.64E-15
GO:0046394	carboxylic acid biosynthetic process	64	9	14.1	2.09	2.29	0.026485
GO:0016053	organic acid biosynthetic process	65	9	13.8	2.06	2.24	0.028981
GO:0006869	lipid transport	60	8	13.3	1.98	2.15	0.046455
GO:0007186	G protein-coupled receptor signaling pathway	158	17	10.8	1.6	1.69	0.036538
GO:1901135	carbohydrate derivative metabolic process	333	35	10.5	1.56	1.66	0.005541
GO:0055114	obsolete oxidation-reduction process	671	69	10.3	1.53	1.65	0.000225
GO:1901564	organonitrogen compound metabolic process	1948	170	8.7	1.3	1.42	9.63E-05
GO:0019538	protein metabolic process	1592	131	8.2	1.22	1.3	0.006493
GO:0008152	metabolic process	3712	282	7.6	1.13	1.23	0.005412

Supplementary Table 2 Gene ontology (GO) of the downregulated genes in *Ago2*$^{-/-}$ mutants as compared to WT mosquitoes at 4 dpi with MAYV.

ID	Name	Bgd count	Result count	Pct of bgd	Fold enrichment	Odds ratio	P-value
GO:0006275	regulation of DNA replication	3	2	66.7	11.01	31.12	0.01054
GO:0009219	pyrimidine deoxyribonucleotide metabolic process	3	2	66.7	11.01	31.12	0.01054
GO:0009221	pyrimidine deoxyribonucleotide biosynthetic process	3	2	66.7	11.01	31.12	0.01054
GO:0009265	2'-deoxyribonucleotide biosynthetic process	3	2	66.7	11.01	31.12	0.01054
GO:0009394	2'-deoxyribonucleotide metabolic process	3	2	66.7	11.01	31.12	0.01054
GO:0019692	deoxyribose phosphate metabolic process	3	2	66.7	11.01	31.12	0.01054
GO:0035278	miRNA-mediated gene silencing by inhibition of translation	3	2	66.7	11.01	31.12	0.01054
GO:0040033	RNA-mediated gene silencing by inhibition of translation	3	2	66.7	11.01	31.12	0.01054
GO:0046385	deoxyribose phosphate biosynthetic process	3	2	66.7	11.01	31.12	0.01054
GO:0006270	DNA replication initiation	12	8	66.7	11.01	31.39	0.00000
GO:0007610	behavior	5	3	60	9.91	23.37	0.00202
GO:0009263	deoxyribonucleotide biosynthetic process	5	3	60	9.91	23.37	0.00202
GO:0018958	phenol-containing compound metabolic process	5	3	60	9.91	23.37	0.00202
GO:0000271	polysaccharide biosynthetic process	4	2	50	8.26	15.56	0.02024
GO:0005978	glycogen biosynthetic process	4	2	50	8.26	15.56	0.02024
GO:0006072	glycerol-3-phosphate metabolic process	4	2	50	8.26	15.56	0.02024
GO:0007064	mitotic sister chromatid cohesion	4	2	50	8.26	15.56	0.02024
GO:0009250	glucan biosynthetic process	4	2	50	8.26	15.56	0.02024
GO:0019748	secondary metabolic process	4	2	50	8.26	15.56	0.02024
GO:0033692	cellular polysaccharide biosynthetic process	4	2	50	8.26	15.56	0.02024
GO:0043044	ATP-dependent chromatin remodeling	4	2	50	8.26	15.56	0.02024
GO:0044550	secondary metabolite biosynthetic process	4	2	50	8.26	15.56	0.02024
GO:0052646	alditol phosphate metabolic process	4	2	50	8.26	15.56	0.02024
GO:0009262	deoxyribonucleotide metabolic process	6	3	50	8.26	15.58	0.00385
GO:0006261	DNA-dependent DNA replication	24	10	41.7	6.88	11.23	0.00000
GO:0007098	centrosome cycle	5	2	40	6.61	10.37	0.03239
GO:0031023	microtubule organizing center organization	5	2	40	6.61	10.37	0.03239
GO:0032392	DNA geometric change	26	10	38.5	6.35	9.83	0.00000
GO:0032508	DNA duplex unwinding	26	10	38.5	6.35	9.83	0.00000
GO:0008643	carbohydrate transport	8	3	37.5	6.19	9.35	0.00984
GO:0005976	polysaccharide metabolic process	6	2	33.3	5.5	7.78	0.04667
GO:0005977	glycogen metabolic process	6	2	33.3	5.5	7.78	0.04667
GO:0006073	cellular glucan metabolic process	6	2	33.3	5.5	7.78	0.04667
GO:0006112	energy reserve metabolic process	6	2	33.3	5.5	7.78	0.04667
GO:0007205	protein kinase C-activating G protein-coupled receptor signaling pathway	6	2	33.3	5.5	7.78	0.04667
GO:0009147	pyrimidine nucleoside triphosphate metabolic process	6	2	33.3	5.5	7.78	0.04667
GO:0035195	gene silencing by miRNA	6	2	33.3	5.5	7.78	0.04667
GO:0044042	glucan metabolic process	6	2	33.3	5.5	7.78	0.04667
GO:0044264	cellular polysaccharide metabolic process	6	2	33.3	5.5	7.78	0.04667
GO:0110154	RNA decapping	6	2	33.3	5.5	7.78	0.04667
GO:0110156	methylguanosine-cap decapping	6	2	33.3	5.5	7.78	0.04667
GO:0140013	meiotic nuclear division	6	2	33.3	5.5	7.78	0.04667
GO:1903046	meiotic cell cycle process	6	2	33.3	5.5	7.78	0.04667
GO:0006606	protein import into nucleus	9	3	33.3	5.5	7.79	0.01410
GO:0034504	protein localization to nucleus	9	3	33.3	5.5	7.79	0.01410
GO:0007062	sister chromatid cohesion	10	3	30	4.95	6.67	0.01925
GO:0051170	import into nucleus	10	3	30	4.95	6.67	0.01925
GO:0006221	pyrimidine nucleotide biosynthetic process	11	3	27.3	4.5	5.84	0.02530

GO:0006220	pyrimidine nucleotide metabolic process	12	3	25	4.13	5.19	0.03225
GO:0006367	transcription initiation from RNA polymerase II promoter	12	3	25	4.13	5.19	0.03225
GO:0006260	DNA replication	68	17	25	4.13	5.28	0.00000
GO:0000280	nuclear division	17	4	23.5	3.89	4.8	0.01684
GO:0017148	negative regulation of translation	17	4	23.5	3.89	4.8	0.01684
GO:0032504	multicellular organism reproduction	17	4	23.5	3.89	4.8	0.01684
GO:0034249	negative regulation of cellular amide metabolic process	17	4	23.5	3.89	4.8	0.01684
GO:0048609	multicellular organismal reproductive process	17	4	23.5	3.89	4.8	0.01684
GO:0000070	mitotic sister chromatid segregation	13	3	23.1	3.81	4.67	0.04008
GO:0016441	posttranscriptional gene silencing	13	3	23.1	3.81	4.67	0.04008
GO:0035194	post-transcriptional gene silencing by RNA	13	3	23.1	3.81	4.67	0.04008
GO:0072528	pyrimidine-containing compound biosynthetic process	13	3	23.1	3.81	4.67	0.04008
GO:0017038	protein import	22	5	22.7	3.75	4.59	0.00892
GO:0140014	mitotic nuclear division	14	3	21.4	3.54	4.25	0.04878
GO:0031327	negative regulation of cellular biosynthetic process	44	9	20.5	3.38	4.03	0.00108
GO:0019953	sexual reproduction	20	4	20	3.3	3.9	0.02971
GO:0044703	multi-organism reproductive process	20	4	20	3.3	3.9	0.02971
GO:0000003	reproduction	30	6	20	3.3	3.9	0.00821
GO:0022414	reproductive process	30	6	20	3.3	3.9	0.00821
GO:0009890	negative regulation of biosynthetic process	46	9	19.6	3.23	3.81	0.00151
GO:0010558	negative regulation of macromolecule biosynthetic process	42	8	19	3.15	3.68	0.00327
GO:2000113	negative regulation of cellular macromolecule biosynthetic process	42	8	19	3.15	3.68	0.00327
GO:0048285	organelle fission	22	4	18.2	3	3.46	0.04079
GO:1901617	organic hydroxy compound biosynthetic process	22	4	18.2	3	3.46	0.04079
GO:0006259	DNA metabolic process	200	32	16	2.64	3.05	0.00000
GO:0010629	negative regulation of gene expression	76	10	13.2	2.17	2.37	0.01588
GO:0031324	negative regulation of cellular metabolic process	76	10	13.2	2.17	2.37	0.01588
GO:0006281	DNA repair	110	12	10.9	1.8	1.92	0.03389
GO:0051276	chromosome organization	194	21	10.8	1.79	1.91	0.00691
GO:0006974	cellular response to DNA damage stimulus	121	13	10.7	1.77	1.88	0.03122
GO:0009892	negative regulation of metabolic process	121	13	10.7	1.77	1.88	0.03122
GO:0048523	negative regulation of cellular process	121	13	10.7	1.77	1.88	0.03122
GO:0071103	DNA conformation change	133	14	10.5	1.74	1.84	0.03036
GO:0033554	cellular response to stress	138	14	10.1	1.68	1.77	0.03977
GO:0048519	negative regulation of biological process	168	16	9.5	1.57	1.65	0.04810
GO:0055085	transmembrane transport	570	52	9.1	1.51	1.6	0.00187
GO:2000112	regulation of cellular macromolecule biosynthetic process	474	42	8.9	1.46	1.54	0.00816
GO:0009889	regulation of biosynthetic process	488	43	8.8	1.46	1.53	0.00823
GO:0031326	regulation of cellular biosynthetic process	486	43	8.8	1.46	1.54	0.00768
GO:0010556	regulation of macromolecule biosynthetic process	484	42	8.7	1.43	1.51	0.01148
GO:0010468	regulation of gene expression	526	44	8.4	1.38	1.45	0.01777
GO:0006996	organelle organization	402	33	8.2	1.36	1.41	0.04572
GO:0031323	regulation of cellular metabolic process	570	45	7.9	1.3	1.35	0.03973
GO:0060255	regulation of macromolecule metabolic process	610	48	7.9	1.3	1.35	0.03621
GO:0019222	regulation of metabolic process	621	49	7.9	1.3	1.35	0.03316
GO:0051716	cellular response to stimulus	836	63	7.5	1.24	1.29	0.03973
GO:0050896	response to stimulus	903	68	7.5	1.24	1.29	0.03390

Supplementary Table 3 Primers.

Gene ID	Primer Name	Sequence (5' to 3')	Purpose
AAEL017251	Ago2-sg6-crR-F	TTAATACGACTCACTATAGGCTGATGCGTCCAAATGCCCAGTT TTAGAGCTAGAAATAGC	gRNA synthesis
	Ago2-sg28-crR-F	TTAATACGACTCACTATAGGGCGTTGCCACTTGCGCGACGGTT TTAGAGCTAGAAATAGC	
	Ago2-sg3-crR-F	TTAATACGACTCACTATAGGCTCCGAGGGTCAATTCCAAGGTT TTAGAGCTAGAAATAGC	
	Ago2-sg25-crR-F	TTAATACGACTCACTATAGGGCACAGCAGCATGTGAAACCGT TTTAGAGCTAGAAATAGC	
/	sgRNA-R	AAAAGCACCGACTCGGTGCCACTTTTTCAAGTTGATAACGGAC TAGCCTTATTTTAACTTGCTATTTCTAGCTCTAAAAC	
AAEL017251	Ago2_PCR-1F	GCAAGATCAAGATTGACAAGC	PCR
	Ago2_PCR-1R	CGAAAGCGAATCCGTGGTTC	
	Ago2_PCR-2F	GCATTCGCATTCGTCGTCACC	
	Ago2_PCR-2R	CTTGACCTCCTGCTCGTAGAC	
	Ago2_PCR-3F	GTCTTCCGGAACTCATCATG	
	Ago2_PCR-3R	GCAACTGATCGGGGTTACAG	
	Ago2_PCR-4F	GCTGCTACCGTCTGCAAGGG	
	Ago2_PCR-4R	AGCGCGTGAACATGTGGCAC	
AAEL023585	Aep53-1_310F_T7	taatacgactcactatagggCCTGATGGACCTTGACGGAG	dsRNA synthesis
	Aep53-1_773R_T7	taatacgactcactatagggTCACCTCGCTGGGAAGTTTC	
AAEL026751	Aep62_287F_T7	taatacgactcactatagggTAAACAAGCTGCCCCAGGAG	
	Aep62_748R_T7	taatacgactcactatagggCTTCGCGGTCCTTACTGGTT	
AAEL012499	AeHis2A_4F_T7	taatacgactcactatagggTCCCAAAAAGGATCCGCCAA	
	AeHis2A_360R_T7	taatacgactcactatagggCAAGGTACTCTTCGGCAGCA	
AAEL026460	AeHis3_40F_T7	taatacgactcactataggGGAAAGGCTCCCCGTAAACA	
	AeHis3_386R_T7	taatacgactcactatagggCGGGCCAGTTGAATGTCCTT	
AAEL003689	AeHis4_29F_T7	taatacgactcactataggGTCTAGGAAAGGGAGGCGC	
	AeHis4_309R_T7	taatacgactcactatagggACCTCCGAAACCGTACAGTG	
AAEL010781	AeAPE1_RNAi_28F	taatacgactcactatagggACAGCGAAGAATGTGGCAGA	
	AeAPE1_RNAi_526R	taatacgactcactatagggTGGTTTCCGCTTTCGCTTTG	
AAEL012339	Aecdk1_RNAi_181F	taatacgactcactatagggCTCCAGCACCACTCGATTGT	
	Aecdk1_RNAi_734R	taatacgactcactatagggCAGCGGGGGAAACTTCTCTT	
AAEL011772	AeRAD50_RNAi_295F	taatacgactcactatagggCGGTCGATGAAGGTTTCCCA	
	AeRAD50_RNAi_724R	taatacgactcactatagggCCTGCCTGCTTTTGTCAACC	
AAEL012960	AeKPNA2_RNAi_435F	taatacgactcactatagggAAACCAAGGCCGTGATCGAT	
	AeKPNA2_RNAi_937R	taatacgactcactatagggCAACGCTTCTTAGGGCTGGA	
AAEL012336	AeEOF1-RNAi-F	taatacgactcactatagggAGCCCGTCCAAGAGGAAGTT	
	AeEOF1-RNAi-R	taatacgactcactatagggCTCGGATGGTACTCACACAA	
AAEL008879	AeKMO-RNAi-271F	taatacgactcactatagggATGAAGGGCCGCATGTTACA	
	AeKMO-RNAi-837R	taatacgactcactatagggCAGATCAATCGCATCGGGGA	
AAEL009273	Aegy_dsIMPDH_F	taatacgactcactatagggGAAGAAGATGTCGCCCTTA	
	Aegy_dsIMPDH_R	taatacgactcactatagggCTCTTGCGTAATGCAGATGG	
AAEL010196	dsTry196_F	taatacgactcactatagggCAATGGTTCGCATCATTCTT	
	dsTry196_R	taatacgactcactatagggTTAATCAGTTGACCACCGGAA	
AAEL000379	CRVP_RNAi_F	taatacgactcactatagggCGCTAATCTACACTTGCGTT	
	CRVP_RNAi_R	taatacgactcactatagggAGCCACCACAATGGAC	
/	EGFP RNAi F2	taatacgactcactatagggTCGACTTCAAGGAGGACGGCA	
/	EGFP RNAi R2	taatacgactcactatagggAAGCGCGATCACATGGTCCT	
AAEL011562	AaeDronc_RT_F	AGCTGGGATTTACCGTGTTC	qPCR
	AaeDronc_RT_R	CGAAACATTCCGTCGATAGG	
AAEL012143	AaeCASPS7_RT_F	CCAACATGGCAAGAAGTACG	
	AaeCASPS7_RT_R	TTCTGCTGGTGCATCATAGG	
AAEL014348	AaeCASPS8_RT_F	AAACGACCTCGATTCTGGTG	

	AaeCASPS8_RT_R	AACCGTTCTTGGTGTTACGG	
AAEL014196	AaeIMP_RT_F	AGCAAACGTTGTTCGAGCTG	
	AaeIMP_RT_R	ACAGGGCGTTGTCTGGATTC	
AAEL002286	AaeATG5_RT_F	GGATGGACAGCAATGGAACT	
	AaeATG5_RT_R	AAGTGGGCCTCAACAATGTC	
AAEL007162	AaeATG8_RT_F	CCCGTTATTGTCGAGAAAGC	
	AaeATG8_RT_R	ATCGTAGCCGATGTTGGTGG	
AAEL009089	AaeATG12_RT_F	ATTCTCCATGCAACGGGTAG	
	AaeATG12_RT_R	TATCTGATCTGGCGATGGTG	
AAEL012143	AaeATG7_RT_F	TGGGGAGCTCAAAACGTATC	
	AaeATG7_RT_R	GCCTTAGGCTTTCCTCCATT	
AAEL023585	Aaep53-1_754F	GAAACTTCCCAGCGAGGTGA	
	Aaep53-1_869R	GGGGAATGATGACCGCTAGG	
AAEL026751	Aaep62_1031F	GTCGTCCGACTTCTGCTTCA	
	Aaep62_1143R	TCCTTCTTGGGGGTTTTCCG	
AAEL026460	AaeHis3_109F	GGAAAGGCTCCCCGTAAACA	
	AaeHis3_233R	TAACGACGGATCTCACGCAG	
AAEL003689	AaeHis4_151F	CCAGGGTATAACGAAGCCCG	
	AaeHis4_251R	CCTTCAGTACGCCTCGTGTT	
AAEL012499	AaeHis2A_315F	ACCTGTTGGCCGAAGTACTG	
	AaeHis2A_448R	GGACACATGACTCAGCAGCT	
AAEL012339	Aecdk1_188F	TTGGACAGTGAAACGGAGGG	
	Aecdk1_219R	GGCTACGTCGAACAGTTCCA	
AAEL011772	AeRAD50_226F	ACCGTTGAAAGCATGGGTCA	
	AeRAD50_343R	CAAACTTGGGTTGTTGGCCC	
AAEL002785	AePOLE2_135F	GCTAGAGGACATCGCCAGTC	
	AePOLE2_235R	CGGTGTTGTTGGCTTCCTTG	
AAEL010781	AeAPE1_795F	GCAAGAGGCAAACCCAAAGG	qPCR
	AeAPE1_934R	TGCGTTGGGTAACTCCATCC	
AAEL010781	AeMSH6_502F	GTGTGGGCTCATCAGAAGCT	
	AeMSH6_615R	CGATTCCGGCACATAAAGCG	
AAEL021206	AeXLF_202F	TACACCGCGGAAATCGTCAA	
	AeXLF_334R	CCGGAATGCTTTGCACGTAG	
AAEL003893	AeXPC_42F	GTCCACCAAAAAGCAGCAGG	
	AeXPC_170R	GCATCTTTATCCCCTCCCGG	
AAEL012336	AeEOF1-qPCR-F	CTGCGGCTTCATGTTCTGTAT	
	AeEOF1-qPCR-R	CTTGCTACATGCCACATTGTG	
AAEL008879	AeKMO-qPCR-870F	CAAGACCAGGCCTCAATCGT	
	AeKMO-qPCR-992R	GCATTCATTCCCTGCCCGTA	
AAEL009273	Aa_RT_IMPDH_F	CCTCAAGCCACTGCTGTGTA	
	Aa_RT_IMPDH_R	ATGCTCCCAAGGAAAGAGCC	
AAEL010196	Try196_RT_F	CGCAAGAGGAATGCCACAAG	
	Try196_RT_R	TTGAATCCAGCGCAGACCAT	
AAEL000379	CRVP_qpcr_F	CGGCACCAAACAGTTCAAG	
	CRVP_qpcr_R	CGCCGTAACGCAAGTGTAG	
AAEL009496	RT rpS7 F	GCAGACCACCATTGAACACA	
	RT rpS7 R	CACGTCCGGTCAGCTTCTTG	

Supplementary Methods

Fitness test

Larval development, pupation time, sex ratio, body weight and adult survival rate

Fifty WT (Liverpool strain) and *Ago2*$^{-/-}$ 1st instar larvae were reared in a container with 800 ml DI water and fish food, and the number of female and male pupae was recorded each day to determine the development time and sex ratio. n=6 biological replicates. Statistical significance between *Ago2*$^{-/-}$ mutants and WT was determined by two-way ANOVA with multiple comparisons.

The whole-body weight of one-day-old females and males was measured by an analytical balance. n=6 biological replicates. Statistical significance between *Ago2*$^{-/-}$ mutants and WT was determined by an unpaired two-sided *t*-test.

Twenty female or male adults were placed into a cup (16 oz) after emergence, and 10% sterile sucrose solution was provided with a cotton ball and changed every 2 days. n=5 biological replicates. The number of dead mosquitoes in each cup was recorded each day until 32 days. *P*-value was determined by a logrank (Mantel-Cox) test.

Blood feeding and percentage of blood feeding

One-week-old females were starved overnight, and twenty of them were weighted and then transferred to a small cup (16 oz). After recovering for 2 h in insectary, mosquitoes were then fed on an anesthetized mouse for 20 min. The blood-fed mosquitoes in each cup were determined as partly and fully engorged and counted. The blood-meal size was calculated by subtracting the weight of the mosquitoes before blood feeding from the weight afterwards, as measured on a fine balance. n=6 biological replicates. Statistical significance between WT and *Ago2*$^{-/-}$ mutants was determined by using an unpaired two-sided *t*-test.

Fecundity and hatching rate

One-week-old females were fed on mice, and fully engorged mosquitoes were sorted. At 3 days post blood-feeding, individual females were each placed in a well of 24-well plate that had 0.5ml of 2% agarose. After 3 days, the females were removed from the plate. The number of eggs in each well was pictured under an inverted microscope and counted. The eggs were kept in insectary for another 4 days and then hatched in DI water, and larvae were counted 3 days after hatching. The hatching rate was

calculated by dividing the number of larvae by the number of eggs laid per female. n=48 biological samples. Any female that laid less than 10 eggs were excluded from the analysis. Statistical significance between WT and *Ago2*$^{-/-}$ mutants was determined by using an unpaired two-sided Mann-Whitney test.

Uncropped western blot images in **Supplementary Fig.9**.

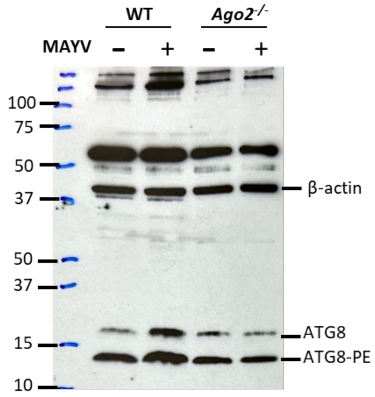

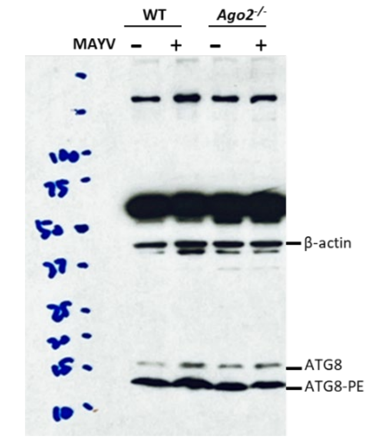

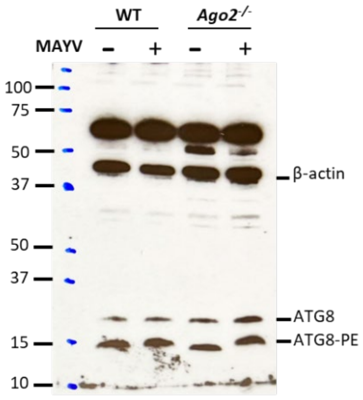

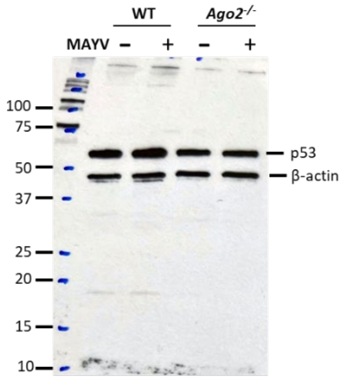

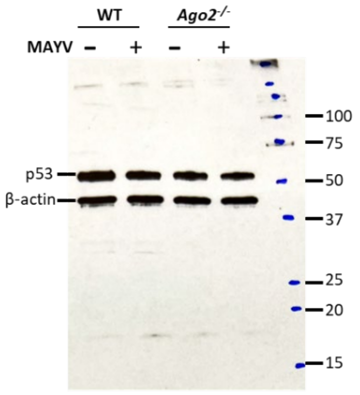

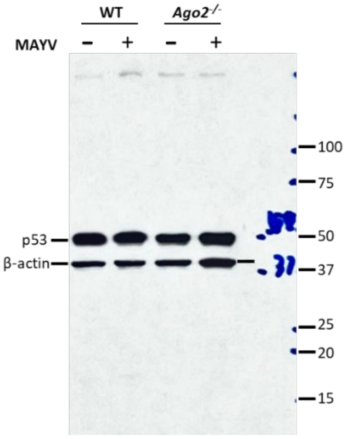

Supplement: Supplementary file 1 — Supplementary Information [file 41467_2023_41370_MOESM1_ESM.pdf]
