# Supplementary material for: Aedes aegypti Argonaute 2 controls arbovirus infection and host mortality
Source: Nat Commun. 2023 Sep 18;14:5773. doi: 10.1038/s41467-023-41370-y (PMC10507101; doi:10.1038/s41467-023-41370-y)
Supplement: Supplementary file 3 — Description of Additional Supplementary Files [file 41467_2023_41370_MOESM3_ESM.pdf]

Description of Additional Supplementary Files

File Name: Supplementary Data 1
Description: Differentially expressed (DE) genes and the transcriptome-wide expression of histone and histone-related, DNA repair and upregulated immunity genes in *Ago2*$^{-/-}$ mutants as compared to WT mosquitoes at 4 dpi with MAYV.

File Name: Supplementary Data 2
Description: Differentially expressed (DE) genes and the transcriptome-wide expression of histone and histone-related, DNA repair and predicated immunity genes in *Ago2*$^{-/-}$ mutants as compared to WT mosquitoes at 4 days post naïve blood feeding.
