# Supplementary material for: Aedes aegypti Argonaute 2 controls arbovirus infection and host mortality
Source: Nat Commun. 2023 Sep 18;14:5773. doi: 10.1038/s41467-023-41370-y (PMC10507101; doi:10.1038/s41467-023-41370-y)
Supplement: Supplementary file 6 — Reporting Summary [file 41467_2023_41370_MOESM6_ESM.pdf]

nature portfolio

Corresponding author(s): George Dimopouls

Last updated by author(s): Aug 20, 2023

Reporting Summary

Nature Portfolio wishes to improve the reproducibility of the work that we publish. This form provides structure for consistency and transparency in reporting. For further information on Nature Portfolio policies, see our Editorial Policies and the Editorial Policy Checklist.

Statistics

For all statistical analyses, confirm that the following items are present in the figure legend, table legend, main text, or Methods section.

n/a	Confirmed	
☐	☒	The exact sample size (*n*) for each experimental group/condition, given as a discrete number and unit of measurement
☐	☒	A statement on whether measurements were taken from distinct samples or whether the same sample was measured repeatedly
☐	☒	The statistical test(s) used AND whether they are one- or two-sided<br>*Only common tests should be described solely by name; describe more complex techniques in the Methods section.*
☐	☒	A description of all covariates tested
☐	☒	A description of any assumptions or corrections, such as tests of normality and adjustment for multiple comparisons
☐	☒	A full description of the statistical parameters including central tendency (e.g. means) or other basic estimates (e.g. regression coefficient) AND variation (e.g. standard deviation) or associated estimates of uncertainty (e.g. confidence intervals)
☒	☐	For null hypothesis testing, the test statistic (e.g. *F*, *t*, *r*) with confidence intervals, effect sizes, degrees of freedom and *P* value noted<br>*Give P values as exact values whenever suitable.*
☒	☐	For Bayesian analysis, information on the choice of priors and Markov chain Monte Carlo settings
☐	☒	For hierarchical and complex designs, identification of the appropriate level for tests and full reporting of outcomes
☒	☐	Estimates of effect sizes (e.g. Cohen's *d*, Pearson's *r*), indicating how they were calculated

Our web collection on statistics for biologists contains articles on many of the points above.

Software and code

Policy information about availability of computer code

Data collection	RNA seq data and small RNA sequencing data.
Data analysis	chopchop CRISPR/Cas9 (https://chopchop.cbu.uib.no/); Vectorbase; ShinyGO (http://bioinformatics.sdstate.edu/go/); Revigo (http://revigo.irb.hr/); Cytoscape v3.9; Usegalaxy (https://usegalaxy.org/); Geneious Prime 2022; GraphPad Prism 10; BioRender.com.

For manuscripts utilizing custom algorithms or software that are central to the research but not yet described in published literature, software must be made available to editors and reviewers. We strongly encourage code deposition in a community repository (e.g. GitHub). See the Nature Portfolio guidelines for submitting code & software for further information.

Data

Policy information about availability of data

All manuscripts must include a data availability statement. This statement should provide the following information, where applicable:

- Accession codes, unique identifiers, or web links for publicly available datasets
- A description of any restrictions on data availability
- For clinical datasets or third party data, please ensure that the statement adheres to our policy

The source data for each graph are provided as a Source Data file. The RNA sequencing and small RNA sequencing data generated in this study have been deposited in NCBI's Sequence Read Archive (SRA) under accession number PRJNA889408. Link: https://dataview.ncbi.nlm.nih.gov/object/PRJNA889408?reviewer=mkrus9rb1iebqokljbkh54kmf2&page=1&sort_by=-release-date.

Research involving human participants, their data, or biological material

Policy information about studies with human participants or human data. See also policy information about sex, gender (identity/presentation), and sexual orientation and race, ethnicity and racism.

Reporting on sex and gender	not applicable
Reporting on race, ethnicity, or other socially relevant groupings	not applicable
Population characteristics	not applicable
Recruitment	not applicable
Ethics oversight	not applicable

Note that full information on the approval of the study protocol must also be provided in the manuscript.

Field-specific reporting

Please select the one below that is the best fit for your research. If you are not sure, read the appropriate sections before making your selection.

☒ Life sciences ☐ Behavioural & social sciences ☐ Ecological, evolutionary & environmental sciences

For a reference copy of the document with all sections, see nature.com/documents/nr-reporting-summary-flat.pdf

Life sciences study design

All studies must disclose on these points even when the disclosure is negative.

Sample size	All the sample sizes are indicated in the methods and figure legends. Here is a summary. For RNAseq and small RNA sequencing, three biological samples were used in light of data reproducibility and the cost. For all the qPCR data, we used 4 biological replicates to minimize the errors. IFA were performed on at least 6 midguts or salivary glands for each sample, three of them were captured under the confocal microscope images, and one of them was shown in the manuscript for each sample. For TEM, we used 3 midguts for each sample and images were captured from all three samples because of time and cost of the TEM. To confirm the data reproducibility, we performed at least two independent replicates for each experiment of virus infection assay, and each replicate had more than 10 individual mosquito sample.
Data exclusions	No data exclusions.
Replication	RNAseq and small RNA sequencing were performed once with three biological replicates. qPCR was performed once with four biological replicates. IFA was performed once with six biological samples. TEM was performed once with three biological samples. Virus infection assay was performed twice with more than biological samples each time. Both attempts at replication were successful.
Randomization	All the samples were allocated randomly.
Blinding	Blinding was not relevant to my study because all the samples were collected with their biological labels by investigators.

Reporting for specific materials, systems and methods

We require information from authors about some types of materials, experimental systems and methods used in many studies. Here, indicate whether each material, system or method listed is relevant to your study. If you are not sure if a list item applies to your research, read the appropriate section before selecting a response.

Materials & experimental systems

n/a	Involved in the study	
☐	☒	Antibodies
☐	☒	Eukaryotic cell lines
☒	☐	Palaeontology and archaeology
☐	☒	Animals and other organisms
☒	☐	Clinical data
☒	☐	Dual use research of concern
☒	☐	Plants

Methods

n/a	Involved in the study	
☒	☐	ChIP-seq
☒	☐	Flow cytometry
☒	☐	MRI-based neuroimaging

Antibodies

Antibodies used	Aedes aegypti anti-ATG8 polyclonal antibody (generated in mouse as polyclonal antibodies, a gift from Dr. Alexandra Raikhel); anti-P53 rabbit polyclonal antibody (ABclonal, cat#: A3185); Anti-β-actin-peroxidase antibody (Sigma, cat#: A5316); Anti-MAYV E2 antibody (Sigma, cat#: MABF3046); mouse hyperimmune ascitic fluid specific for DENV2 (keep in the lab from the previously published work).
Validation	Anti-ATG8 polyclonal antibody: a gift from Dr. Alexandra Raikhel. It was originally produced by Bryant et al. 2011 and had been confirmed to its reactivity to Aedes aegypti ATG8 in the same study. (PLoS One. 2011;6(11):e25502. doi: 10.1371/journal.pone.0025502.)<br>Anti-P53 rabbit polyclonal antibody and β-actin-peroxidase antibody: Western blot were performed to confirm its reactivity with Aedes aegypti P53 or actin based on the predicated molecular weight of the protein. Data provided in the manuscript.<br>Anti-MAYV E2 antibody: Antibody specificity to Mayaro viruses was validated by ELISA on the website (https://www.sigmaaldrich.com/US/en/product/mm/mabf3046). Its reactivity to viruses in mosquitoes was tested in this study by IFA through comparing MAYV infected and uninfected mosquitoes.<br>Mouse hyperimmune ascitic fluid specific for DENV2: It was generated and confirmed its reactivity to dengue viruses by Xi et al. (2008) and kept at -80oC freezer in the same lab. Original publication is PLOS Pathogens 4(7): e1000098 (https://doi.org/10.1371/journal.ppat.1000098).

Eukaryotic cell lines

Policy information about cell lines and Sex and Gender in Research

Cell line source(s)	C6/36 (ATCC); BHK 21 (ATCC); Vero (ATCC).
Authentication	None of the cell lines used were authenticated.
Mycoplasma contamination	BHK 21 and Vero cells were tested negative for mycoplasma contamination and cultivated with ant-mpp (Invivogen). C6/36 cells were not tested for mycoplasma contamination.
Commonly misidentified lines (See ICLAC register)	None.

Animals and other research organisms

Policy information about studies involving animals; ARRIVE guidelines recommended for reporting animal research, and Sex and Gender in Research

Laboratory animals	BALB/c mice (6 to 8 weeks old) were used for mosquito blood feeding. Mice were reared at 27°C under 50% relative humidity with a 12h light/12h dark cycle. Aedes aegypti strains were used: exu-Cas9, Liverpool, Ago2-/- and AgoN-/-, and were kept at 27°C under 85% relative humidity with a 12h light/12h dark cycle.
Wild animals	The study did not involve wild animals.
Reporting on sex	The mice were used only for mosquito blood-feeding, and gender-based analysis was not relevant to the study.
Field-collected samples	The study did not involve samples collected from the field.
Ethics oversight	This study was carried out in accordance with the recommendations in the Guide for the Care and Use of Laboratory Animals of the National Institutes of Health, the Animal Care and Use Committee (ACUC) of the Johns Hopkins University, and the institutional Ethics Committee (permit number: M006H300). The IACUC committee approved the protocol.

Note that full information on the approval of the study protocol must also be provided in the manuscript.
